# Automated age grading of female *Culex pipiens* by an optical sensor system coupled to a mosquito trap

**DOI:** 10.1186/s13071-024-06606-w

**Published:** 2024-12-18

**Authors:** María I. González Pérez, Bastian Faulhaber, Mark Williams, Joao Encarnaçao, Pancraç Villalonga, Carles Aranda, Núria Busquets

**Affiliations:** 1 IRTA. Programa de Sanitat Animal. Centre de Recerca en Sanitat Animal (CReSA), Campus de la Universitat Autònoma de Barcelona (UAB), Bellaterra, Catalonia Spain; 2 Unitat Mixta d’Investigació IRTA-UAB en Sanitat Animal. Centre de Recerca en Sanitat Animal (CReSA). Campus de La Universitat Autònoma de Barcelona (UAB), Bellaterra, Catalonia Spain; 3 Irideon S.L, Barcelona, Spain; 4 Servei de Control de Mosquits del Consell Comarcal del Baix Llobregat, El Prat del Llobregat, Catalonia Spain

**Keywords:** *Culex pipiens*, Mosquito vectors, Age grading, Chronological age, Optical sensor, Machine learning

## Abstract

**Background:**

The age distribution of a mosquito population is a major determinant of its vectorial capacity. To contribute to disease transmission, a competent mosquito vector, carrying a pathogen, must live longer than the extrinsic incubation period of that pathogen to enable transmission to a new host. As such, determining the age of female mosquitoes is of significant interest for vector-borne diseases surveillance and control programs.

**Methods:**

In this contribution, an automated age-grading system was developed to classify the age of female *Culex pipiens*, which is the primary vector of West Nile virus and other pathogens of medical and veterinary importance in northern latitudes. The system comprises an optical wingbeat sensor coupled to the entrance of a mosquito trap and a machine learning model. Three age classes were used in the study: young (2–4 days), middle (7–9 days) and old (14–16 days). From a balanced dataset of flight data, four features were extracted: wingbeat fundamental frequency, spectrogram, power spectral density and Mel frequency cepstral coefficients. The features were used for training with the XGBoost algorithm to generate a model for age classification.

**Results:**

The best performing model was trained with the power spectral density feature on two age classes, ≤ 4 days old and ≥ 7 days old, and had an accuracy of 71.8%.

**Conclusions:**

An automated mosquito age-grading system was applied for the first time to our knowledge for automated age classification in mosquitoes; and complements the mosquito genus and sex classification capability of the system reported in our previous work. The system may find use in mosquito-borne disease surveillance and control to help to discriminate young mosquitoes (≤ 4 days old) from older mosquitoes, which may act as vectors of arboviruses.

**Graphical Abstract:**

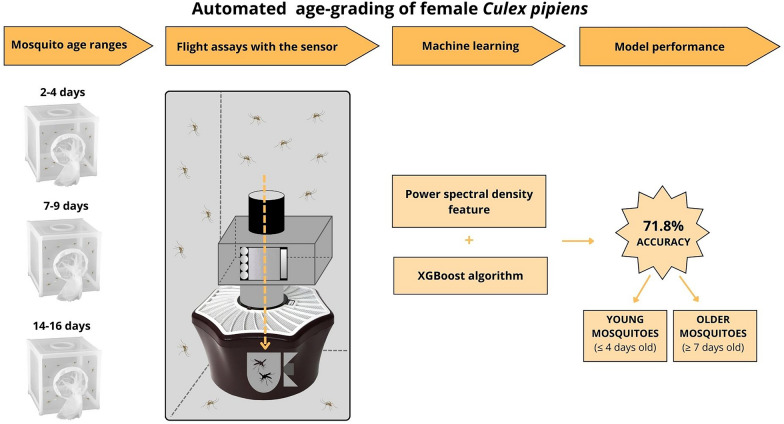

## Background

*Culex* (*Culex*) *pipiens* Linnaeus, 1758, also known as the “common house mosquito” or “Northern house mosquito,” is a polytypic species and member of a species complex which is now distributed worldwide. It is native to Africa, Asia and Europe, where it is the primary vector of important human and animal pathogens such as West Nile virus (WNV), Usutu virus, Sindbis virus, Tahyna virus, lymphatic filariasis and avian malaria [1]. Females of *Cx. pipiens* feed on a variety of vertebrate hosts, contributing to the amplification of the enzootic cycle of arboviruses such as WNV, which is sustained mainly among wild birds, with occasional spillover to humans [2]. Preventing the spread of mosquito-borne diseases (MBD) and responding to outbreaks is a priority for public health authorities, which are developing coordinated strategies to strengthen vector control programs worldwide [3].

The age distribution of a mosquito population is a key determinant of its vectorial capacity (VC) to transmit a pathogen to a host [4]. To become a vector, an adult female mosquito must live longer than the extrinsic incubation period (EIP) of the pathogen that it carries, which ranges from several days to a couple of weeks depending on factors such as vector population, pathogen load and ambient temperature. For example, the EIP of WNV in *Cx. pipiens* is typically > 7 days at 18 to 27 ºC [5–8]. As such, the impact of MBD can be reduced by vector control strategies which target adult mosquito lifespan [9].

The importance of vector longevity for the epidemiology and control of MBD was first described in the 1950s by Macdonald as a key component of the basic reproduction rate of malaria [10]. Following this, the concept of VC was introduced, which indicates the ability of a population of vectors to transmit a pathogen to a host [11]. The VC is calculated as $VC=\frac{{ma}^{2}{bp}^{n}}{-ln\left(p\right)}$ where ‘m’ is the vector density relative to host density; ‘a’ is the probability a vector feeds on a host in one day; ‘b’ is the competence of the vector for a particular virus; ‘p’ is the daily probability of the vector survival; ‘n’ is the EIP in days. A linear reduction in ‘p’ leads to an exponential reduction in VC, which highlights the impact of vector survivorship on VC [12].

Several methods have been described in the literature to estimate mosquito age [12]. One of the oldest and most established of these is based on the examination of changes in the ovarian morphology of female mosquitoes according to their reproductive status, such as the ovary tracheation method or the determination of sequential egg laying events [13]. The posterior finding of age-related changes in particular biochemical signals in mosquitoes, such as the pteridines or the cuticular hydrocarbons, led to the development of biochemical methods which aimed to quantify these components by chromatography [14]. In the last decades, the introduction of molecular methods such as transcriptional and protein profiling contributed to the advancement of mosquito age grading by analyzing the differences in the expression levels of age-responsive genes and proteins [15]. More recently, the use of near-infrared and mid-infrared spectroscopy has served to identify specific mosquito biological traits like age based on the quantification of changes in the absorption spectra of organic compounds in the exoskeleton [16].

Despite decades of research and their epidemiological relevance as vectors, only a few age grading methods have been developed for *Culex* species [17–19]. These methods, based on ovarian dissection and cuticular hydrocarbons, are either labor-intensive, require a high level of expertise and complex equipment and processing, or do not directly provide an estimation of chronological age (calendar days) [12]. To address these limitations, a new age-grading method for female *Cx. pipiens* mosquitoes was developed and assessed in the present work. This method consisted of a system comprising an optical wingbeat sensor coupled to a mosquito trap and a machine learning (ML) model, which provides an automated classification of mosquitoes by age.

Several studies have previously investigated the potential of different type of sensing devices (acoustic, optical, image based) in combination with ML to identify mosquitoes and other attributes of mosquito biology [20, 21]. Optical (also known as optoacoustical) sensors, like the one presented in the current work, profit from insect bioacoustic properties, which have been under study for classification purposes since the first half of the twentieth century [22–24]. In the last decade, the number of published works using optical sensing approaches to classify flying mosquitoes by genus, species, sex or parity status has increased [25–31]. However, age classification using such devices has not been attempted before, even though mosquito wingbeat frequency has been reported to vary with age [32].

In the present contribution, the authors attempted to close the gap in age-grading methods for *Culex* species by proposing a new technological approach consisting of an optical wingbeat sensor coupled to a mosquito trap. This method was previously tested in both laboratory and field conditions to classify *Aedes* and *Culex* mosquitoes by genus and sex, reporting high-accuracy results [33, 34]. In this case, the sensor was trained with *Culex* female mosquito samples of different ages to build a ML model for age classification. This is the first time this kind of system has been applied for mosquito age classification to our knowledge. We hope this work opens new possibilities in this field, with a view to its future application in vector biology research and in mosquito surveillance and control programs.

## Methods

### Mosquito rearing conditions

Larvae of *Cx. pipiens*, population of Bellaterra, Cerdanyola del Vallés, Barcelona, Spain (2020 and 2022), were kept in plastic trays containing 750 ml dechlorinated tap water renewed three times per week and fed with fish food pellets (Goldfish Sticks-TETRA, Melle, Germany). Pupae were placed in plastic cups inside insect-rearing cages (BugDorm-1 Insect Rearing Cage W30 × D30 × H30 cm, MegaView Science, Talchung, Taiwan) until adult emergence. Adult female mosquitoes were anesthetized with carbon dioxide in a plate (Flowbuddy flow regulator, 59-122BC, Flystuff, California, USA) and sorted into three age classes: young (2–4 days), middle (7–9 days) and old (l4–16 days). They were fed 10% sucrose solution ad libitum; this was removed 24 h before the flight assay for a particular age class.

The mosquito life cycle took place inside a climatic chamber (Telewig, Barcelona, Spain) at 28 ºC, 80% relative humidity, with a light:dark photoperiod of 11:11 h (plus 1 h of dusk and 1 h of dawn). Different generations of adult mosquitoes were used to obtain samples for the experiment until F15. All female mosquitoes used in the experiment were nulliparous.

### Flight assays using the optical sensor and mosquito trap

An optical wingbeat sensor (Irideon, Barcelona, Spain) was coupled to the entrance of a BG-Mosquitaire mosquito trap (Biogents, Regensburg, Germany) containing a suction fan. The sensor comprises an optical emitter formed by a two-dimensional array of light-emitting diodes that emit a collimated light beam (940 nm) toward an optical receiver formed by a two-dimensional photodiode array with a sensing zone formed between them. When a mosquito flies close to the entrance of the sensor, it is likely to be sucked into the sensor by the airflow of the fan and pass through the sensing zone where it casts a fast-changing shadow on the optical receiver because of the modulation of the light beam by the wingbeats of the mosquito in flight. A detailed description of the sensor is provided in our previous works [33, 34].

**Fig. 1:**
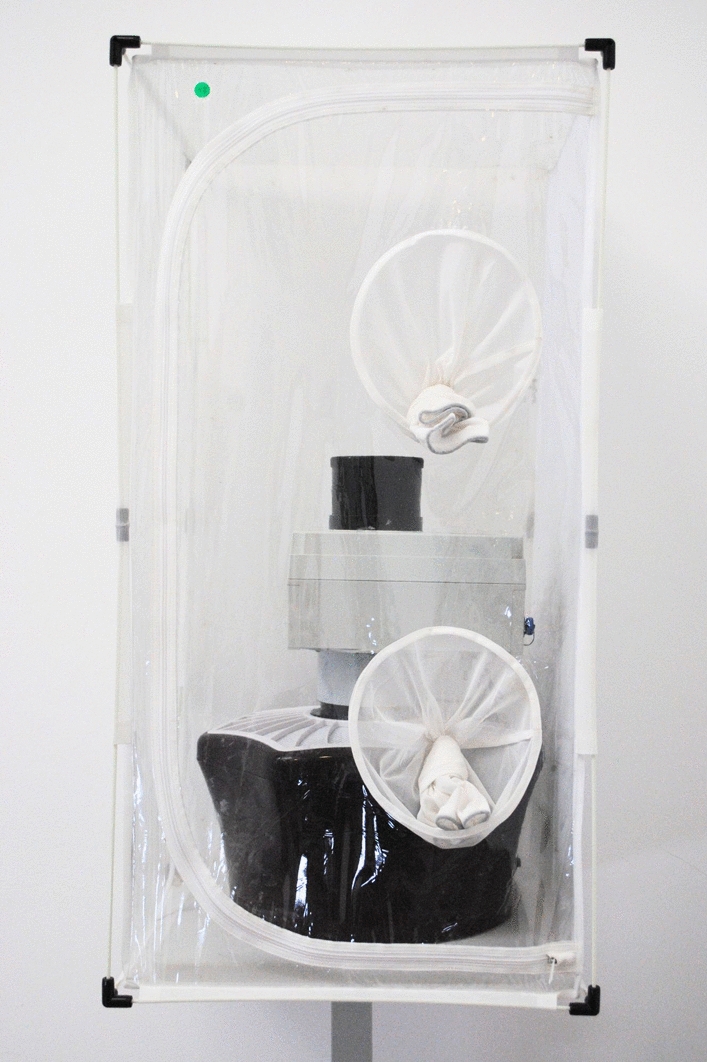
Experimental setup for the flight assays

The sensor and trap were placed in an insect cage (BugDorm-4S4590 W47.5 × D47.5 × H93.0 cm, MegaView Science, Talchung, Taiwan) inside a climatic chamber (CCK-0/5930 m, Dycometal, Barcelona, Spain) where the flight assays took place (Fig. 1). The trap was fitted with a sachet of BG-Sweetscent (Biogents, Regensberg, Germany) to attract mosquitoes toward the sensor. During each flight assay, female mosquitoes belonging to a particular age class were released into the insect cage. Flight assays were performed at ambient temperatures of 18 ºC, 23 ºC and 28 ºC, and mosquitoes were acclimatized in the climatic chamber for 24 h prior to the start of the assay.

### Machine learning model for age classification

The sensor recordings were downloaded to a laptop computer after each flight assay and were then processed using a Python script. Pre-processing of data included the manual examination of each recording and the exclusion of those considered to be unrepresentative, e.g. single recordings with two mosquitoes or recordings where the mosquito was deemed to have hit the wall of the transparent flight tube inside the sensor. The resulting labeled data were randomly undersampled to obtain a balanced dataset which was split, with 75% used to train the supervised ML model, with fourfold cross validation, and the remaining 25% used to test the classification performance of the model on unused data. A series of wingbeat features, including wingbeat fundamental frequency (WBF), spectrogram, power spectral density (PSD) and Mel frequency cepstral coefficients (MFCC), were extracted from the sensor recordings and used to train the ML model with the XGBoost gradient boosting algorithm [35]. For every feature, training was done using fourfold cross-validation, and the model with the best cross-validation score was then selected for testing. The performance of the ML model was assessed using the accuracy metric, which was calculated by dividing the number of correct predictions by the total number of predictions.

**Fig. 2:**
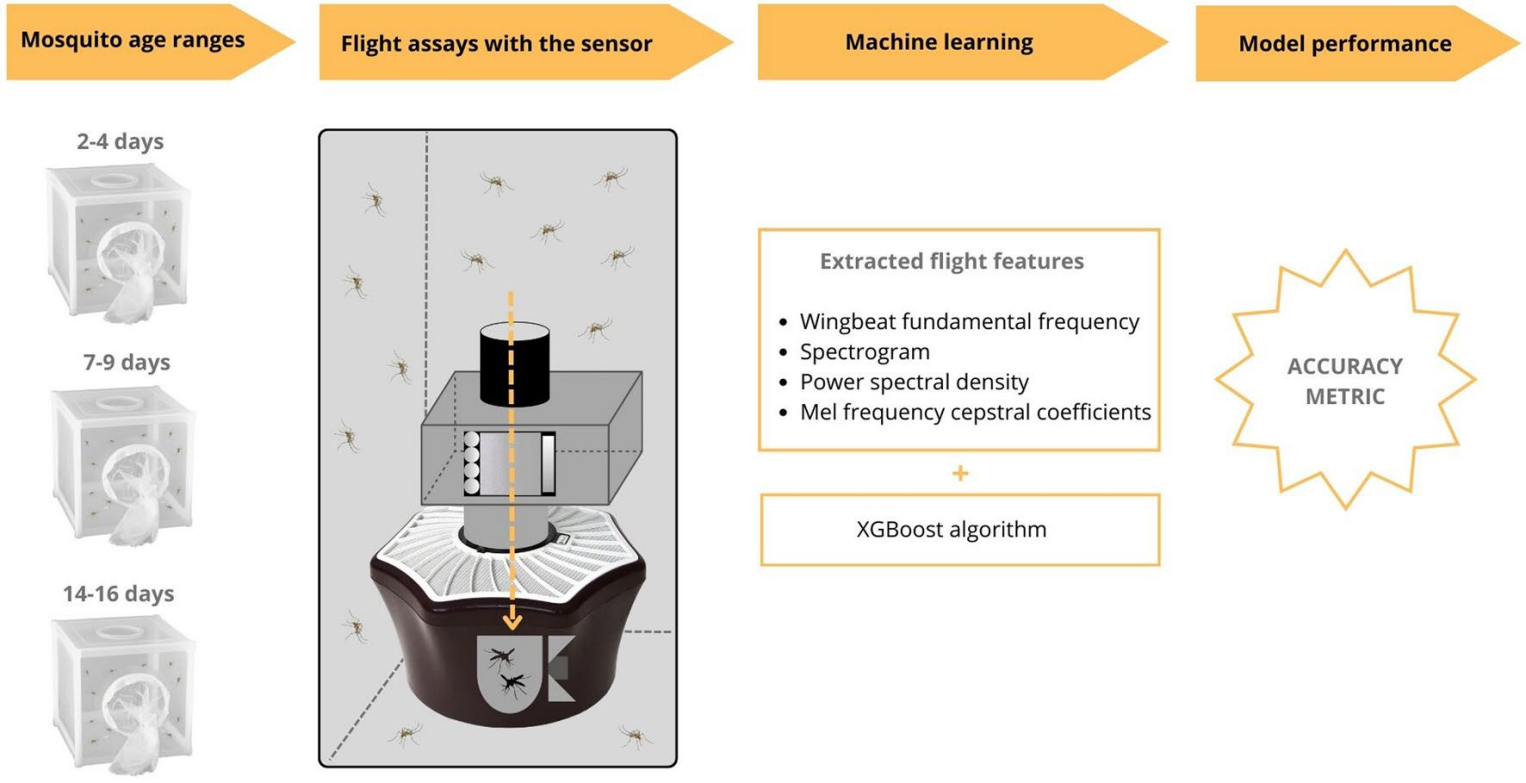
Diagram illustrating the study methodology

A schematic diagram of the whole methodological procedure is illustrated in Fig. 2.

## Results

The balanced dataset used for training and test contained a total of 2088 recordings of female *Cx. pipiens* from the three age classes: young (2–4 days), middle (7–9 days) and old (14–16 days).

A first ML model based on the gradient boost algorithm was trained to classify mosquitoes into the three age classes. The four features, WBF, PSD, spectrogram and MFCC, were evaluated, and the best performing feature was the PSD with an accuracy of 46.6%. The low accuracy result for this model is largely due to the confusion between the middle and old age classes, as shown in the confusion matrix (Table 1).

**Table 1:** Confusion matrix for ML classification into three age classes, young (2–4 days), middle (7–9 days) and old (14–16 days), using the PSD feature

Actual age	Predicted age		
	2–4 days	7–9 days	14–16 days
2–4 days	**95**	37	42
7–9 days	41	**74**	59
14–16 days	31	69	**74**

Considering the accuracy results of the first model in Table 2 and the high level of confusion between the middle and old age classes, a second model was trained with the middle and old age classes combined into a single “older” class. This model was also based on the gradient boost algorithm and classified into only two age classes: young (2–4 days) and older (7–16 days). In this case, the balanced dataset contained a total of 1392 recordings. The same four features were evaluated, and the best performing feature continued to be PSD, but with an improved accuracy of 71.8% (Table 2). A training score of 100% indicated that these results could be improved further by using more training data.

**Table 2:** Accuracy for the different wingbeat features for ML classification into three age classes and two age classes

Feature	Accuracy of classification	
	Three age classes [young (2–4 days), middle (7–9 days) and old (14–16 days)]	Two age classes [young (2–4 days) and older (7–16 days)]
PSD	46.6%	71.8%
MFCC	46.2%	68.7%
Spectrogram	44.6%	65.8%
WBF	37.0%	53.5%

*PSD* power spectral density, *MFCC* Mel frequency cepstral coefficients, *WBF* wingbeat fundamental frequency

## Discussion

In this contribution, a new method was described to determine the age of female *Cx. pipiens* mosquitoes. This method consisted of an optical wingbeat sensor coupled to a mosquito trap with an ML model to classify age classes based on mosquito wingbeat features. To the best of our knowledge, this is the first publication of results for an automated age classification system based on a wingbeat sensor.

We note that the same automated classification system was used in previous laboratory [33] and field studies [34] for genus and sex classification of *Aedes* and *Culex* mosquitoes with high accuracy. While other optical sensor systems have been used to classify flying insects (by genus, species, sex or parity status) with good results [25–31], there have been no previous reports of automatic age classification using such sensors.

In insect bioacoustics, the WBF has been traditionally used as a predictor variable for different classification purposes [20]. However, it was demonstrated to be insufficient for certain classification tasks (i.e. taxonomical classification), because of the existence of overlapping frequency distributions among different mosquito species [25]. Therefore, many studies have chosen other more complex acoustic features as predictors, such as the spectrogram, PSD or MFCC, which provide better classification results [28–31].

Previous research with *Aedes aegypti* reported that the WBF increased significantly with age [32], although this increase only took place at young ages and plateaued for older ages [36]. In the present study, using WBF as the sole predictor variable for age classification gave the lowest accuracy due to the overlap between age classes; the richer spectrogram, MFCC and PSD features all gave better accuracy results. The MFCC and PSD features were also used in our prior work [33] and in other mosquito classification studies based on bioacoustic sensing [29, 37–39]. In the present study, the widely used ML algorithm XGBoost was used because it gave good accuracy results for mosquito classification in other works [29] and in our prior work [33, 34].

The best age classification model, which gave an accuracy of 71.8%, used the PSD feature to distinguish young (2–4-days old) from older (7–16-days old) mosquitoes. As indicated by the training score metric, the accuracy could possibly be improved using more training samples.

In the present work, the optical sensor system was able to differentiate between young mosquitoes (≤ 4 days old) and older mosquitoes. However, it was less able to different between the middle and old ages, which may be due to the lack of change in the features used. In line with vector competence studies of *Cx. pipiens* for WNV [5], this young-older binary classification may serve to discriminate between two functional groups of non-vector and potential vector mosquitoes which are old enough to have overcome the EIP of the virus. The EIP of WNV in *Cx. pipiens* has been reported to be > 7 days at temperatures ranging from 18 to 27 ºC [5–8]. Therefore, this method, if properly calibrated for field applications, could be useful to assess WNV control interventions targeting vector longevity.

As described, the sensor-based system should enable automated age grading of wild female *Culex* mosquitoes in the field, with potential application to other mosquito vectors, which would be very significant for vector surveillance and control programs. The results indicate that the accuracy of the ML model could still be improved by using more laboratory flights to train the model.

## Conclusions

Mosquitoes of *Culex* genera are one of the main vectors worldwide. The age distribution of such mosquito populations is a key determinant of their vectorial capacity since only those mosquitoes which have lived enough to overcome the EIP of a pathogen will be able to transmit it to a new host. In this article, we assessed a new method for age grading of female *Cx. pipiens* based on an automated system using an optical wingbeat sensor coupled to a mosquito trap with a machine learning model. The results derived from the present work could be useful in mosquito-borne disease surveillance and control to help to discriminate young (≤ 4 days old) from older mosquitoes which may act as vectors of arboviruses.

## Data Availability

Data supporting the main conclusion of this study are included in the manuscript. The datasets analyzed during this study are available from the corresponding author on reasonable request.

## Abbreviations

EIP: Extrinsic incubation period
MBD: Mosquito-borne diseases
MFCC: Mel frequency cepstral coefficients
ML: Machine learning
PSD: Power spectral density
VC: Vectorial capacity
WBF: Wingbeat fundamental frequency
WNV: West Nile virus
